# Changes after cancer diagnosis and return to work: experience of Korean cancer patients

**DOI:** 10.1186/s12885-021-07812-w

**Published:** 2021-01-21

**Authors:** Ka Ryeong Bae, Juhee Cho

**Affiliations:** 1 Department of Clinical Research Design & Evaluation, Samsung Advanced Institute of Health Sciences & Technology (SAIHST), Sungkyunkwan University, 115 Irwon-ro, Building C 5F, Rm. 27, Gangnam-gu, Seoul, South Korea; 2 Cancer Education Center, Samsung Medical Center, Seoul, South Korea; 3 Center for Clinical Epidemiology, Samsung Medical Center, Seoul, South Korea

**Keywords:** Cancer survivors, Return to work, Interview, Qualitative research

## Abstract

**Background:**

Cancer patients’ return to work is a growing aspect of survivorship care, yet limited studies have been conducted in Korea to understand the work-related experience of cancer patients. The purpose of this study was to understand the unmet needs of cancer patients and identify the necessary factors to develop a vocational intervention program based on cancer patients’ work-related experience after cancer diagnosis.

**Methods:**

Semi-structured individual in-depth interviews were conducted with 50 cancer patients who were working at the time of diagnosis at a university hospital in Seoul, South Korea from July to September of 2017. Interview data were analyzed using qualitative content analysis.

**Results:**

‘The changes patients experienced after cancer diagnosis’ were categorized into Personal and socio-environmental changes. ‘Personal changes’ were changes within the patient that were further divided into ‘physical’, ‘psychological’ and ‘spiritual’ changes while ‘socio-environmental changes’ were changes in either ‘attitude’ and ‘relationship’ of other people cancer patients encountered. In addition to these post-diagnosis changes, the following 4 major factors related to return-to-work were identified to affect patients’ experience: ‘fear of cancer recurrence’, ‘financial status’, ‘informational support’, and ‘job-related work environment’.

**Conclusion:**

Cancer patients’ working status was determined by personal and socio-environmental changes after the cancer diagnosis which as well as psychological distress and practical issues such as fear of cancer recurrence, financial burden, and work environment. Educational materials and intervention programs informing patients on these changes and factors may facilitate their return-to-work after diagnosis.

**Supplementary Information:**

The online version contains supplementary material available at 10.1186/s12885-021-07812-w.

## Background

The efforts for cancer prevention, early detection, and advanced health care technology have recently increased the 5-year cancer survival rate from 42.9 to 70.4% during 1993–1995 and today, there are over 1.86 million cancer survivors in South Korea [1]. Thus, a much larger number of cancer patients diagnosed with cancer are expected to become long-term survivors who combine routine and work.

Despite the difference in cancer types and the social welfare system, approximately 49–91% of cancer patients return to work within a year worldwide [2–7]. However, according to the analysis of the national health insurance data, about half of all working cancer patients lose their jobs after receiving a cancer diagnosis, and only up to 40% of them are reemployed [8, 9]. This may be attributed to various reasons, but the key influencing factors seem to be: gender, age, occupation type, level of income [9], medical conditions including chemotherapy [10], and comorbidities [11]. Furthermore, the level of understanding from co-workers, social support system available to the patient, information sharing [12], the prevailing stigma, and the presence/absence of discrimination toward cancer patients [13] have also been suggested to affect patients’ work-related decisions. However, there are very few studies in Korea that reflect the socio-cultural factors of Korea on the difficulties associated with returning to work for cancer patients.

The importance surrounding a cancer patient’s return to work has long been recognized, and various methods have been applied at the level of the hospital, region, or nation in establishing intervention programs that could help cancer patients return to work. These include programs which minimize the physical challenges endured during cancer treatment, while providing knowledge concerning the patient’s return-to-work, as well as programs with a diversity of contents regarding psychological-educational, physical, and occupational rehabilitation [14]. However, only a handful of studies have evaluated the effects of the intervention programs and have reported a statistically significant result. This is because the measured variables were inappropriate for evaluating the programs, or the interventions had not been helpful in the actual return to work of the patients [14, 15].

Qualitative studies in Korea that examine cancer patients’ work-related experiences after receiving their diagnoses are limited to breast cancer patients [12, 16], or to the experience of a small number of cancer patients [17–19]. Therefore, a thorough understanding of cancer patients’ work-related experiences cannot be achieved based on previous studies – as such, it is necessary that further studies be conducted regarding cancer patients’ experiences. Qualitative studies should first be conducted on cancer patients in the Korean context so as to accurately analyze their experiences in relation to work after having received a cancer diagnosis, as well as the difficulties that most frequently arise in such a situation.

The purpose of the study is to explore and understand the cancer patients’ work-related experiences after receiving a cancer diagnosis, and to describe the findings thereof. The key question for this study is “What experiences do cancer patients undergo in relation to their work after cancer diagnosis?”

## Methods

### Study design

This study takes the form of a qualitative content analysis, based on individual in-depth interviews involving cancer patients who used to work at the time of their cancer diagnosis. A qualitative content analysis allows us to explore the patients’ work-related experiences after having received their cancer diagnosis.

### Study subjects

The inclusion criteria for participants were decided to include the following: cancer patients aged 19–64 years; patients who had partook in economic activity as an employee or self-employed at the time of their cancer diagnosis; and patients with an ability to communicate fluently in Korean. The exclusion criterion was decided to encompass all individuals who are unable to participate in an in-depth interview due to a cognitive disability.

The participants were recruited using various methods, including via: a poster attached to the notice board at the outpatient center of the Comprehensive Cancer Center of a university located in Seoul; a post on the website of the Cancer Education Center; an introduction by the attending physician or nurse, based on purposive sampling in cases where the cancer patient has been confirmed to have a record of employment or self-employment at the time of their diagnosis.

### Ethical considerations

This study has been conducted following the approval of the institutional review board at the hospital to which the researchers belong (IRB No. 2017–05-166). Prior to the study, all participants were given a detailed explanation of the study and voluntary written consent was collected. For ethical protection of the participants, the data related to the interview contents were used strictly for study purposes only, while the information was coded for security reasons – both of these measures were explained to each participant. The contents of all interviews were recorded using an audio-recorder. Each participant was notified that the recording would begin at the start of the interview and that all recorded data would be transcribed. Participants were also notified that the recorded data would be discarded completely at the end of the study and that the quotations in the study would not indicate the identity of the participant in any way. The explanation also included the fact that the participant could withdraw from the study at their will and that no harm or disadvantage would be incurred by their withdrawal from the study. Upon completion of the interview, the participant was given a small gift of appreciation for participating in the study.

### Data collection

The individual in-depth interviews used in this study were conducted between July 14 and September 26, 2017. All interviews were conducted by author KB in the consultation room at the hospital with which the patients were familiar with. This one-time semi-structured interview lasted approximately 50 min. Prior to the interview, the researcher provided the patient with a questionnaire containing 11 items; the questionnaire assisted in determining the participant’s basic personal information, as well as their occupation at the time of receiving their cancer diagnosis, their past work experience, and whether they were currently working. At the start of the interview, each patient was again notified that the interview would be recorded from the beginning. Meanwhile, and throughout the entirety of the interview, KB made notes regarding the patient’s linguistic and paralinguistic expressions, the atmosphere, and any other important matters which may not be conveyed via the audio recording alone.

The interviews consisted of semi-structured questions based on the above-mentioned questionnaire, which was developed via a literature review and professional advice. The questions were selected according to the patient’s circumstances and the study’s purpose. For the complete list of questionnaire, please refer to Additional File 1. At the beginning of the interview, the mood was softened through conversation concerning the patient’s daily life and current treatment; the interview then began with an open, extensive question – such as: “Please feel free to tell me what experiences you had, in relation to the work you used to have, after receiving your cancer diagnosis.” The questions focused on the patient’s own thoughts regarding: work; having to work while receiving treatment; undergoing the process of sick leave; suspension or resignation from work; and reemployment, as well as how people reacted or changed, and how the patient subsequently dealt with them. At the end of the discussion, and to close the interview, the patient was asked if he or she could describe the feelings they had experienced during the interview and to share any further stories. Author KB completed the transcription of the interview within 1 or 2 days so as to record the atmosphere and all relevant feelings at the time of the interview as accurately as possible.

### Data analysis

In this study, the interview materials and the notes made during the interview for each patient were transcribed on a computer at the end of each interview so as to ensure a regular cycle of data collection and analysis. All collected data were processed for qualitative content analysis, following the procedure suggested by Elo and Kyngäs [20].

After transcription, authors KB and JC read through the interviews for a general understanding. Thereafter, and through repeated readings, the data were categorized based on meaningful units, and the keyword or sentence for each unit was searched for, followed by open coding. The categorization based on the similarities and differences between each type of coding led to the grouping of larger and more meaningful units; by integrating those units, the final abstract theme was derived. Through ten subsequent meetings– where both researchers checked the data of one another and discussed and converged the points for which the opinions varied – the final result of the data analysis was produced.

### Verification on the trustworthiness of the information

As a qualitative study, the trustworthiness of information in this investigation was increased through complying with the criteria of qualitative studies as suggested by Shenton [21]. First, to increase their credibility, the interviews were led by author KB alone. Both authors KB and JC performed the analysis and the results of the subsequent data analysis were discussed with two other experts (a nurse and a researcher) on the topic of cancer patients in developing a consensus. Authors KB and JC had built an adequate level of background knowledge on the interview questions through the review of numerous previous studies published both in Korea and abroad regarding the occupation and return to work of cancer patients, making it possible for them to collect the data with a sufficient degree of theoretical sensitivity to context and meaning. Second, to increase the transferability of data, 50 patients of different age, gender, cancer type, and working status after their cancer diagnosis were interviewed and each group consisted of a minimum of five patients. For each group, data were collected until saturation was reached. Third, to increase the dependability and confirmability of the results, the data collection and analysis procedures were described in detail, and care was taken to avoid interpreting the interview conditions and experiences within the subjective frame of the researchers. Although the transcripts were not returned to the participants, peer examination, a well-known technique in qualitative research, was used to verify the trustworthiness and credibility of the data [22].

### Researcher training and preparation

Author KB, PhD and a senior researcher at a Comprehensive Cancer Center of a tertiary hospital, is a cancer-specialized nurse with 15 years of experience in clinical studies concerning cancer. Author JC, PhD, the head of Cancer Education Center and associate professor, has conducted numerous survivorship-related research including those of developing educational materials. They have a rich, in-depth understanding of the field, have given lectures on qualitative studies at a graduate school level, participated in seminars and conferences on qualitative studies, and reviewed a large body of books and articles on the methodology, developing a consequent expert level of competence in performing qualitative studies.

## Results

### General characteristics of the subjects

The general characteristics of all 50 participants (at the time of the interviews) were as follows: patients had an average age of 45.72 years; 26 were male (52.0%) and 24 were female (48.0%); 41 were married (82.0%); and 39 had an undergraduate degree or above (78.0%). No participant refused to participate or dropped out of the study. For clinical characteristics, the most frequent cancer type was breast cancer – experienced by 19 patients (38.0%) – followed by 12 patients with lung cancer (24.0%) and 9 patients with lymphoma (18.0%); the most common stage of cancer recorded was Stage 0 and 1 – in 21 patients (42.0%) – and Stage 3 – in 17 patients (34.0%). On average, 20.46 months had passed since the respondents’ cancer diagnosis. For occupational characteristics, the number of patients with a job (at the time of the interview) was the largest – at 26 (52.0%). For the type of job, white-collars accounted for the largest number at 39 (78.0%). The average number of working years was 15.72 (Table 1).

**Table 1:** Characteristics of All Participants

	(***N*** = 50)
**Characteristics**	**N (%)**
**General Characteristics**	
**Age (years),** mean (SD)	45.72 (7.51)
**Sex**	
Male	26 (52.0)
Female	24 (48.0)
**Marital status**	
Married	41 (82.0)
Single/ divorced/ separated/ widowed	9 (18.0)
**Highest Level of Education received**	
≤ High school	11 (22.0)
≥ College	39 (78.0)
**Monthly Income (10,000 KRW)**	
< 500	27 (54.0)
≥ 500	23 (46.0)
**Clinical Characteristics**	
**Type of Cancer**	
Breast cancer	19 (38.0)
Liver cancer	4 (8.0)
Lung cancer	12 (24.0)
Lymphoma	9 (18.0)
Others	6 (12.0)
**Stage**	
0/I	21 (42.0)
II	7 (14.0)
III	17 (34.0)
IV	2 (4.0)
Unknown	3 (6.0)
**Time since diagnosis (months),** mean (SD)	20.5 (22.80)
**Occupational Characteristics**	
**Current Employment Status**	
Continued working	26 (52.0)
Changed jobs	1 (2.0)
Leave of absence	16 (32.0)
Resigned	7 (14.0)
**Type of Job**	
Blue-Collar	4 (8.0)
Sales or Service	5 (10.0
White-Collar	39 (78.0)
Self-employed	2 (4.0)
**Working years,** mean (SD)	15.72 (10.92)

*SD* Standard deviation, *KRW* Korean Won

### Analysis results

**Fig. 1:**
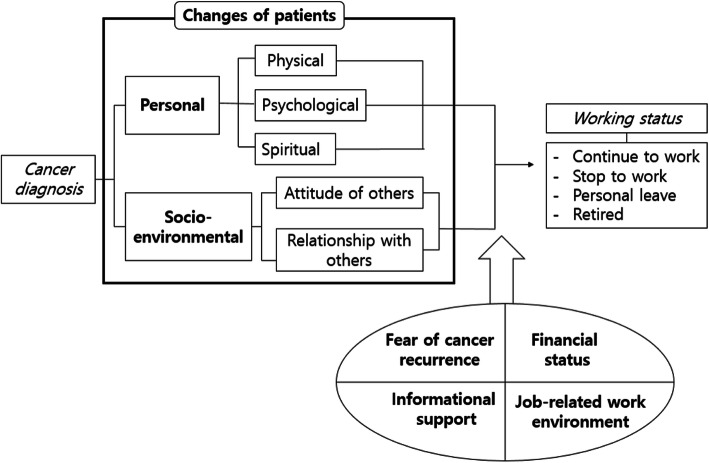
Conceptual framework of changes after cancer diagnosis and return to work

The participants’ work-related experiences after receiving their cancer diagnosis could be divided into two categories, six subcategories, and 17 codes (as presented in Table 2). The categorical structure is shown in Fig. 1. The results show that patients experienced personal and socio-environmental changes due to cancer and along with these changes, four factors affecting the decision to return to work modified participants’ ultimate decision to continue working or not, whether the latter meant taking a leave of absence or permanently leaving the job.

**Table 2:** Work-Related Experiences of Cancer Survivors

Categories	Subcategories		Codes
Changes after cancer diagnosis	**Personal changes**	Physical	Changes in appearance
			Physical weakness (fatigue)
			Physical discomfort (pain, numbness, nausea, vomiting, lymphedema etc.)
		Psychological	Identity changes
			Mood changes
		Spiritual	Meaning of work / Changes in priority
	**Socio-environmental changes**	Attitude of others	Showing excessive support and consideration
		Relationship with others	Maintaining social relationships
Factors influencing the return to work	**Fear of cancer recurrence**		Anxiety, uncertainty
			Work-related stress
	**Financial status**		Being financially responsible for the family
			Burden of health cost
	**Informational support**		Unverified information/Lack of available information
	**Job-related work environment**		Uncertainty of employment
			Lack of substitute workers
			Flexibility of work
			Support of workplace

### Category I: changes after cancer diagnosis

After being diagnosed with cancer, participants were found to have undergone two main changes regarding work-related experiences; personal and socio-environmental changes. For each change, there were cases where the change had a substantial impact on increasing the patient’s challenge to continue working, and cases where the change had a negligible impact on the patient – posing no significant challenge to maintaining their previous work.

#### Personal changes

##### Physical changes

Among the changes experienced by cancer patients, physical changes included physical discomfort caused by the treatment of cancer. Firstly, changes in appearance – such as hair loss, changes in skin tone and complexion, and weight loss – are shown to have contributed to the resignation or avoidance of returning to work for many female patients, and was the most significant factor in preventing return-to-work for service workers and patients with tasks that require a considerable number of face-to-face encounters. Second was the reduction in physical strength due to fatigue, which was complained as the most substantial physical change experienced by all cancer patients participating in this study. Patients commonly reported being easily exhausted for tasks they had engaged in before cancer diagnosis. Being in such vulnerable physical state reduced patients’ perceived work ability, satisfaction, and performance and as a result, their quality of working life and work-related stress were negatively affected. Third and last physical change patients experienced from receiving treatment were changes due to treatment side effects – such as pain, numbness in hands and feet, lymphedema, nausea, and vomiting. Experiencing these physical changes ultimately led participants to permanently leave work or take a leave of absence and even for those patients who continued to work, such changes exerted a negative impact on patients’ ability to work, nonetheless.

*My weight went down from 74kg to 52kg after surgery. I lost a lot of fat and muscle so when I tried getting back to my daily routine, I got exhausted even after working an hour or two. I could not get many things done. I was overwhelmed with fatigue whenever I tried to concentrate on something. (Participant 21, HCC, continued working)*

##### Psychological changes

The psychological changes were divided into two domains of changes in personal identity and emotional changes. In some cases, patients would place the stigma of a ‘cancer patient’ on him or herself, subsequently feeling devalued and diminished, regardless of their past career. In this context, the patient contemplated the potential disadvantages that may incur when he or she disclosed the news, thus avoiding telling anyone.

*I just thought I might receive unnecessary stares, [such] as, ‘Is she like that because she can’t take care of her/himself?’, ‘Is she able to get the work done as she did before?’, ‘Is she backing out even when she could do more?’ I mean... you sort of have it labeled as a cancer patient who always makes things difficult for others. (Participant 26, breast cancer, resigned)*

For emotional changes, the sense of social isolation, in particular, was noted upon as the patient viewed him or herself as a ‘good-for-nothing person’ and felt ‘distanced from the society’ after having stopped the work he or she had been engaged in. In many cases, the patient also had a feeling of ‘frustration’ or ‘despair’ that developed into the thought ‘My life is over now’, causing depression and torpor.

*If I leave work, I become a quitter just because of an illness. My work is half of my life. Leaving work would add on to the already existent frustration or despair, you know. The thought of my life being over would grow and to a degree, I would feel as if I've become a good-for-nothing person, you know. Out of touch with society, that sort of feeling. (Participant 23, breast cancer, continued working)*

##### Spiritual changes

In experiencing a serious situation such as a cancer diagnosis, patients were given time to deeply reflect on the work he or she had been doing so as to evaluate the true meaning and values of the work. This is a part of the concept of workplace spirituality [23] and, in the experience thereof, patients underwent a process of transformation regarding their understanding of the meaning of work and the values of life after cancer, when work used to fill the largest portion of their life. As in the case of Participant 20, work used to be the most fulfilling aspect of life in the past; but now, living a healthy life through ‘letting go of desires’ and ‘coordinating aspects regarding their work’ was the main goal in life.

*How others thought of me was important to me. I think I used to want things to be done the way I had decided it to be; I wanted specific outcomes, and that [would lead] others [to] see me as a perfectionist. But from now on, I want to let go of desires and coordinate aspects regarding work. I used to have too many desires. That probably was what deteriorated my health.* (*Participant 20, breast cancer, on leave of absence)*

#### Socio-environmental changes

##### Recognizing the changes in the attitude of others

Many patients felt burdened about their co-workers being overly considerate and regarding them as ‘cancer patients who needed to be helped and taken care of’. At first, cancer patients accepted it as a support for themselves and felt grateful, but as it was taken too much, they regretted even disclosing their diagnosis to co-workers.

*I greatly appreciated my co-workers for showing support and consideration after being diagnosed with cancer. But it wasn’t that pleasant since I was being treated as a patient from the very first start in all these circumstances and gatherings. All the ‘you can’t do this or that because you are a cancer patient’ made me feel alienated from everything. If I hadn’t told them about being diagnosed with cancer, things would have been the way they were.. more comfortable so I regret it. (Participant 25, breast cancer, on leave of absence)*

##### Recognizing the changes in their relationship with others

The most significant changes in the relationship with others at work was shown to originate from the decrease in, or disappearance of, the time shared with others– such as lunch or dinner with co-workers, company dinners, or get-together events after work. What used to be a close relationship either diminished or fell apart, and the patients said they experienced social exclusion, such as being purposely ignored at meetings or being excluded or isolated from the team.

*Now, whatever task they ask me to do, they do seem to feel uncomfortable, and... well... when there is a company dinner or something... it feels like... a bit... that I am being excluded. Before, we used to drink together and everything, but now... I am a cancer patient, so... Ah, no drinking, you know, so... it feels like I'm being excluded from everything. (Participant 14, lung cancer, continued working)*

### Category II: factors influencing the return to work

In experiencing various changes, cancer patients face a number of critical factors that influence their decisions regarding whether or not to continue working. Four such factors were found in this study, as follows:

#### Fear of cancer recurrence

The fear of cancer recurrence was expressed via anxiety, uncertainty, and task-related stress. The respondents seemed to think that, as they returned to work in a physical condition that differed from the past, they would overwork or get more stressed, which may at any time cause the recurrence of cancer. Thus, even while working or planning to return to work, patients found themselves reluctant to focus on their work as they had done before, given the fear of undergoing cancer again, which was thus recognized as a significant factor.

*[There is] a lot of work to do... and frankly, my body is not as healthy, and if something goes wrong, cancer could recur, and these thoughts continue to haunt me day after day. ‘Am I going to die from the pressure and stress I get from working as I did before cancer?’ It almost feels hopeless. (Participant 40, rectal cancer, resigned)*

#### Financial status

Most of the male respondents, in particular, had a main goal of maintaining a livelihood as the breadwinner who ‘must take financial responsibility for the family,’ as in the case of Participant 41. In addition, patients without a spouse had numerous reasons for continuing, or starting, to work – for the purpose of maintaining a livelihood.

*If I had a lot of money, I wouldn't have returned to the company. I have to earn, anyhow, so that I can financially support my family without difficulty. If I take time off for too long, so that I fail to readily adapt to the work and quit in the end, then I will have to look for a new job, and I'll be caught in a vicious circle. These reasons, along with the thought that I should stay employed for a long time, drove me to return to work rather early. (Participant 41, lymphoma, continued working)*

Despite the relatively large benefits received from both the Exempted Calculation of Health Insurance and private insurance in regard to the medical costs, frequent visits to the hospital – with the consequent expenses of transportation, food, and lodging – were viewed as a significant financial burden to respondents. In particular, the place where this study was conducted is a large general hospital in Seoul, the capital city of South Korea, and many of the patients had to take an express train from a provincial region for consultations and appointments, thereby requiring the need to find lodging and food while staying close to the hospital for the treatment, adding on to the already existent financial difficulties.

*I come from Mokpo, and the transportation alone costs 120,000 won ($100). But for the treatment, I only pay 1,700 won ($1.50); then, I eat something and take the high-speed train nearby hospital... You see, the time and money spent for coming to Seoul is much more. (Participant 7, lung cancer, continued working)*

#### Informational support

Above all, cancer patients expressed their frustration at the extremely low level of informational support with respect to cancer and work. Regarding work, the respondents reported feeling confused while sharing non-professional content acquired from the Internet or TV programs in an indiscreet way, rather than being provided with accurate and credible information. Even at the hospital – their only source of professional information – there was a general lack of educational materials or programs and, in many cases, patients found it difficult to obtain accurate information on cancer and work from the medical staff who were busy throughout the day.

*There are so many things I need to know such as how to live everyday life, what to eat, what to avoid considering my health status. But everyone gives me different information. No one knows it for sure. And, well … the doctor says I could eat anything but some patients do not believe it. (Participant 41, thyroid cancer, continued working)*

The easy access to the Internet in Korea has led many cancer patients to use online communities in order to exchange information with other patients with the same type of cancer and to receive substantial psychological comfort. In particular, the patients who had returned to work would connect to the Internet at any time to get the information in regard to being a cancer patient at work, to share the burden with other patients of similar jobs, and to ask and answer each other’s questions. However, almost no work-related information could be obtained from the medical staff at the hospital. Most patients spoke of an experience – as in the case of Participant 3 – where the medical staff was too busy to give him or her a chance to ask about work, and when they did ask about returning to work, the answer was brief and without any concern for the type of work or the tasks involved; ‘you can work’ or ‘you can return to work’ was all they received; as such, patients felt uncertain whether it really was alright to return to work.

*The doctor didn't ask, and I had no chance to ask. They come in, and it's over in three minutes. They are busy, I know, but it's just [that] you don’t have enough time or the conscience to ask anything in that short window of time... not enough time for just consultations either, because it's just the way it is... I have so many questions but what can I do? I just go to [an] Internet community to leave questions and get a lot of information there. (Participant 3, lung cancer, on leave of absence)*

#### Job-related work environment

After being diagnosed with cancer, the greatest concern patients had was the uncertainty of employment. Even when it is guaranteed, receiving a negative performance assessment led to disadvantages in salary or promotion. Thus, some patients had to return to work immediately after surgery or never express the hardship they were going through during treatment. Some patients had lost their jobs after notifying the company of their cancer diagnosis and were, subsequently, job-seeking while in the middle of treatment.

Due to the lack of substitute workers, it was highly difficult for cancer patients to use sick leave or a leave of absence for their treatment. Although the sudden cancer diagnosis, and the onset of treatment like surgery, made it inevitable, patients were sometimes criticized for being ‘irresponsible’ at work, as in the case of Participant 13. The pressure from co-workers regarding the extra work they had to take from lack of substitute workers aggravated the circumstance.

*I was totally stressed out right up to the moment I took my leave of absence. This is, well... they didn't make it easy for me. You have to work, they said, because when you’re gone, another person has to take on your workload. That was their reaction. They told me that I was irresponsible. (Participant 13, breast cancer, on leave of absence)*

The flexibility of work was a significant requisite for cancer patients who frequently visited the hospital for treatment and status examination. In some cases, it was possible to adjust the working hours until full recovery or to freely take an afternoon off or a day off for the chemotherapy or radiotherapy, or to take additional leave of absence or request for childcare leave – although these options were rarely available without restriction.

*I wanted to take a leave of absence, but it was limited to one year. So, if I wanted to take it for more than a year, I had to leave my job. There needs to be a more flexible policy for sick leave that considers patients’ treatment and severity of illness. (Participant 19, breast cancer, on leave of absence)*

## Discussion

In this study, we conducted individual in-depth interviews with 50 cancer patients to understand their work-related experiences after the diagnosis. Patients experienced personal and socio-environmental changes after the cancer diagnosis. Factors such as “fear of cancer recurrence,” “financial status,” “informational support,” and “job-related work environment” affected their return to work. While Korea is a medically advanced country, there are no educational materials or intervention for working cancer patients which could guide them after cancer diagnosis regarding their work. Moreover, cancer patients faced difficulties in disclosing their disease status and continued working due to negative attitudes toward cancer patients. Male patients were more likely to return to work compared to female patients as they felt more financial burden especially if they were the only source of income in their household. Most previous studies that evaluated cancer patients’ returning-to-work experiences after cancer diagnosis found that physical and psychological changes due to cancer diagnosis and treatment made cancer patients difficult to return to work. Patients commonly experienced lymphedema, cognitive dysfunction, fatigue, hair loss, anxiety, and stress [12, 16–19, 24, 25] and such changes negatively affected patients on maintaining the job and performing usual tasks often resulting in changing roles, reduced working hours, taking a leave of absence, and stop working [25]. Similarly, our study participants also told that physical and psychological changes such as pain, altered appearance, and decreased energy made them reduce working hours and stop working. Interestingly none of our study participants reported difficulties due to cognitive dysfunction. This might be due to different types of cancer and study population. While most previous studies were conducted with early-stage breast cancer patients, we included patients with various cancers with a wide age range. While these physical and psychological changes were the most influential factor which affected cancer patients’ return to work, most of them are predictable and manageable thanks to the advances of supportive cancer. Yet, cancer patients need to be informed about possible physical and psychological changes and its potential impact on their work to keep working during and after cancer treatment. Our study participants reported that they did not receive work-related information at either hospitals or communities. Unfortunately, there is no educational material or intervention for working cancer patients even though the five-year survival rate is over 70.4% in Korea [1]. This might be because cancer care in Korea is more focused on treatment (removing a tumor from the body) rather than taking a holistic approach. Considering the meaning of work for patients, it is necessary to develop educational materials and interventions for working cancer patients. According to a recent systematic review, while there have been different types of interventions for helping cancer patients return to work such as psycho-education, physical training, and medical intervention, the effectiveness of these interventions was uncertain. The reviewer suggested that multi-disciplinary intervention could be effective to enhance cancer patients’ experience of return to work [26].

In our study, the attitudes of others, especially those of co-workers affected cancer patients’ working status after diagnosis. Although cancer patients receive the most support from their co-workers [27], they also felt uncomfortable, as the co-workers were too deeply involved in their daily lives. Study participants reported support from their co-workers often felt like a burden and lack of privacy and caused more stress as they had to respond to the support. This might be because of the Korean culture of goodwill. In Korea, people pay attention to others’ life events and personal issues such as cancer diagnosis as they believe it is the way to care for others. However, such attention did not help cancer patients return to work but gave them more emotional distress to disclose cancer diagnosis and continue to work [12]. According to a recent study on co-workers of cancer patients, co-workers expressed difficulties in working with cancer patients as they did not know how to help or behave with them in the light of their co-workers’ emotional struggles [28]. This implies the need for developing an intervention program that can help both the cancer patient and their co-workers at work. A program that teaches how to communicate with co-workers diagnosed with cancers would be necessary for cancer patients and their co-workers. Finally, educating cancer patients and their co-workers will help more cancer patients to return to work and maintain a healthy relationship with their co-workers.

While many cancer patients stopped working right after cancer diagnosis, some patients tried to keep jobs even with heavy treatment schedule because of financial situation. Patients need to work for paying immediate medical cost and making a living. This was especially true for patients whose income was the major source of household income. In our study, male patients felt more financial burden compared to female patients, and they felt more pressure to continue to work. This is interesting considering the financial burden of medical cost in Korea is relatively low compared to those in other countries as most of the cancer treatments cost is covered by the national health insurance. This might be because of the paternalistic Korean culture which emphasizes that the father is responsible for the family’s living expenses. While more women are at the workplace and share household income, this might be true to cancer patients in our study whose mean age is around 60 years.

Similar to the results of previous studies, fear of cancer recurrence was a challenge cancer patients faced when they continue to work. The participants in this study said that, despite having overcome the changes incurred after beating cancer and working as before, the anxiety that the cancer may recur at any time, and the uncertainty thereof, were the challenges they faced while at work. According to a previous study, cancer patients became empowered when they return to work after overcoming fear of cancer recurrence [29]. Multi-disciplinary intervention for helping cancer patients to overcome fear of cancer recurrence and return to work would be necessary. Lastly, the stability of employment, the presence of substitute workers, the flexibility of work, and the support present at work, were found to have a significant influence on a cancer patient’s decision to return to work or continue working. These consist with the previous studies [24, 30]. The institutional support – including sick leave and holiday for health management, flexible working hours, and a facility for repose – as well as reinforced supportive health care policies, should be established to help cancer patients maintain their quality work following reemployment. The solutions for the work environment should also be established, so as to further prevent occupational damages or accidents following the reemployment of cancer patients.

In this study, the participants encompassed cancer patients at a single Comprehensive Cancer Center located in Seoul, the capital of South Korea. As such, their monthly income and level of education were relatively high, with 80% of respondents being made up of specialized office workers. It is, thus, possible that the results do not reflect the cases of extreme hardship with respect to financial and psychological burdens. Nonetheless, interviews were conducted on a large number of cancer patients with a cancer type. Based on the results, this study determined the changes and challenges related to work experienced by cancer patients in Korea after having received a cancer diagnosis. Furthermore, through the comparison with non-Korean studies, similarities and differences were identified. As a result, the significance of this study lies in that possible supports a level of the hospital, workplace, and government have been identified, so as to ensure that cancer patients can continue to work after being diagnosed with cancer in Korea.

## Conclusion

Cancer patients’ working status was determined by personal and socio-environmental changes after the cancer diagnosis which as well as psychological distress and practical issues such as fear of cancer recurrence, financial burden, and work environment. Educational materials and intervention programs informing patients on these changes and factors may facilitate their return-to-work after diagnosis. Future studies would be necessary with more diverse cancer patients at different settings. This current study was a precedent to a quantitative survey examining work-related and other unmet needs of cancer patients. The results of both qualitative and quantitative studies are planned to be used for developing an intervention to facilitate cancer patients’ return-to-work.

## Supplementary Information

**Additional file 1: Supplementary Table 1**. Semi-structured Interview Questions

## Data Availability

The datasets used and analyzed during the current study are available from the corresponding author on reasonable request.

## References

[1] Hong S, Won YJ, Park YR, Jung KW, Kong HJ, Lee ES (2020). Cancer statistics in Korea: incidence, mortality, survival, and prevalence in 2017. Cancer Res Treat.

[2] Pearce A, Tomalin B, Kaambwa B, Horevoorts N, Duijts S, Mols F (2019). Financial toxicity is more than costs of care: the relationship between employment and financial toxicity in long-term cancer survivors. J Cancer Surviv.

[3] Nekhlyudov L, Walker R, Ziebell R, Rabin B, Nutt S, Chubak J (2016). Cancer survivors' experiences with insurance, finances, and employment: results from a multisite study. J Cancer Surviv.

[4] Su M, Zhang N, Cai Y, Wang J, Anderson R, Yao N (2019). Work and income changes after cancer in rural China: a cross-sectional survey. Cancer Med.

[5] Ghasempour M, Shabanloei R, Rahmani A, Jafarabadi MA, Abri F, Khajehgoodari M (2020). The relation of readiness for return to work and return to work among Iranian Cancer survivors. J Cancer Educ.

[6] Lear-Claveras A, Ubalde-Lopez M, Serra Saurina L (2019). Labor market situation after an episode of sickness absence due to malignant neoplasia. Evidence from a Spanish cohort. BMC Public Health.

[7] Endo M, Haruyama Y, Muto G, Imai Y, Mitsui K, Mizoue T (2019). Recurrent sick leave and resignation rates among female cancer survivors after return to work: the Japan sickness absence and return to work (J-SAR) study. BMC Public Health.

[8] Choi KS, Kim EJ, Lim JH, Kim SG, Lim MK, Park JG (2007). Job loss and reemployment after a cancer diagnosis in Koreans - a prospective cohort study. Psychooncology..

[9] Park JH, Park EC, Park JH, Kim SG, Lee SY (2008). Job loss and re-employment of cancer patients in Korean employees: a nationwide retrospective cohort study. J Clin Oncol.

[10] Tamminga SJ, Verbeek J, Bos M, Fons G, Kitzen J, Plaisier PW (2019). Two-year follow-up of a multi-Centre randomized controlled trial to study effectiveness of a hospital-based work support intervention for Cancer patients. J Occup Rehabil.

[11] Chen YY, Wang CC, Wu WT, Lai CH, Ho CL, Hsu YY (2020). Trajectories of returning to work and its impact on survival in survivors with oral cancer: a 5-year follow-up study. Cancer..

[12] Heo EK, Kang HS, Kim KH, Hong YP (2011). Return-to-work experiences among breast cancer survivors. J Korean Soc Matern Child Health.

[13] Cho J, Smith K, Choi EK, Kim IR, Chang YJ, Park HY (2013). Public attitudes toward cancer and cancer patients: a national survey in Korea. Psychooncology..

[14] Bae KR, Cho J, Jeon SH (2019). A literature review of return-to-work interventions for cancer survivors. Korean J Occup Health Nurs.

[15] Lamore K, Dubois T, Rothe U, Leonardi M, Girard I, Manuwald U (2019). Return to work interventions for Cancer survivors: a systematic review and a methodological critique. Int J Environ Res Public Health.

[16] Kim JS (2016). Return-to-work experience of breast Cancer survivors: a qualitative study based on grounded theory approach [dissertation].

[17] Son M, Lee JS (2015). Return to work experience among military officers with cancer. J Korean Acad Nurs.

[18] Kim MH, Kim JS, Kim HN (2016). Return-to-work experiences among nurses after receiving cancer treatment. JKAIS..

[19] Lee IJ (2019). A phenomenological approach to the job-seeking experience of Cancer survivors in low-income. Health Soc Welfare Rev.

[20] Elo S, Kyngas H (2008). The qualitative content analysis process. J Adv Nurs.

[21] Shenton AK (2004). Strategies for ensuring trustworthiness in qualitative research projects. Educ Inf.

[22] Janesick VJ. Peer Debriefing. Blackwell Encyclopedia Sociol. 2015. 10.1002/9781405165518.wbeosp014.pub2.

[23] Ashmos D, Duchon D (2000). Spirituality at work: a conceptualization and measure. JMI..

[24] Stergiou-Kita M, Grigorovich A, Tseung V, Milosevic E, Hebert D, Phan S (2014). Qualitative meta-synthesis of survivors' work experiences and the development of strategies to facilitate return to work. J Cancer Surviv.

[25] Butow P, Laidsaar-Powell R, Konings S, Lim CYS, Koczwara B (2020). Return to work after a cancer diagnosis: a meta-review of reviews and a meta-synthesis of recent qualitative studies. J Cancer Surviv.

[26] de Boer AG, Taskila TK, Tamminga SJ, Feuerstein M, Frings-Dresen MH, Verbeek JH. Interventions to enhance return-to-work for cancer patients. Cochrane Database Syst Rev. 2015. 10.1002/14651858.CD007569.pub3. 10.1002/14651858.CD007569.pub3 PMC6483290 26405010

[27] Islam T, Dahlui M, Majid HA, Nahar AM, Mohd Taib NA, Su TT (2014). Factors associated with return to work of breast cancer survivors: a systematic review. BMC Public Health.

[28] Yoon J, Park SM, Kim IR, Lee JE, Lee S, Yoo JH (2018). Experiences and difficulties when working with Cancer survivors. KJPO..

[29] Allen JD, Savadatti S, Levy AG (2009). The transition from breast cancer 'patient' to 'survivor'. Psychooncology..

[30] Pryce J, Munir F, Haslam C (2007). Cancer survivorship and work: symptoms, supervisor response, co-worker disclosure and work adjustment. J Occup Rehabil.

