# Supplementary material for: Changes after cancer diagnosis and return to work: experience of Korean cancer patients
Source: BMC Cancer. 2021 Jan 21;21:86. doi: 10.1186/s12885-021-07812-w (PMC7818925; doi:10.1186/s12885-021-07812-w)
Supplement: Supplementary file 1 — Additional file 1: Supplementary Table 1. Semi-structured Interview Questions [file 12885_2021_7812_MOESM1_ESM.docx]

Supplementary Table 1. Semi-structured Interview Questions

| Core question | No. | Sub-questions |
| --- | --- | --- |
| Describe your work-related experience after cancer diagnosis or during treatment. | 1.1 | How would you describe your work activity since cancer diagnosis? |
|  | 1.2 | Did you take a leave of absence during treatment? |
|  | 1.3 | How did your family members, colleagues and manager/supervisor  respond to you retaining your job? |
|  |  | How did your family members, colleagues and manager/supervisor  respond to you returning to work? |
|  | 1.4 | Did your company make any accommodations for you regarding  working hours, job tasks and others? |
|  | 1.5 | What difficulties did you encounter after returning to work? |
|  |  | What difficulties do you expect to encounter when returning to work? |
| How much does your employment affect your family and/or livelihood activities? | 2.1 | Do you have a family to support?  Do you have a family to support you? |
|  | 2.2 | How much is your work activity responsible for your family living? |
|  | 2.3 | How do you pay for your treatment? |
|  | 2.4 | Do other members of your family financially contribute to the family? |
| Are you receiving any support to maintain your work activity or return-to-work? | 3.1 | Where do you get information related to your work activity?  Where do you get information necessary for return-to-work? |
|  | 3.2 | Are you receiving any financial support for your treatment or work activity? |
|  | 3.3 | What information or support do you think are necessary for return-to-work? |
| Do you have any other work-related experience after cancer diagnosis? | 4.1 | Do you want to return to work? |
|  | 4.2 | Did you share your cancer diagnosis with your colleagues and managers?  How did they respond to the news? |
|  | 4.3 | Describe, if any, a positive experience you had at work after cancer diagnosis. |
